# Quercetin promotes the proliferation, migration, and invasion of trophoblast cells by regulating the miR‐149‐3p/AKT1 axis

**DOI:** 10.1002/kjm2.12887

**Published:** 2024-08-20

**Authors:** Dan Wang, Xin‐Rui Zhao, Yi‐Fan Li, Rui‐Lin Wang, Xue‐Bing Li, Chun‐Xia Wang, Yong‐Wei Li

**Affiliations:** ^1^ The Second Clinical Medical College Henan University of Chinese Medicine Zhengzhou City Henan Province China; ^2^ Chinese Medicine College Hong Kong Baptist University Kowloon City Hong Kong China; ^3^ Department of Clinical Laboratory Centre Henan Province Hospital of Traditional Chinese Medicine, The Second Affiliated Hospital of Henan University of Chinese Medicine Zhengzhou City Henan Province China

**Keywords:** AKT1, miR‐149‐3p, quercetin, recurrent spontaneous abortion

## Abstract

Recurrent spontaneous abortion (RSA) has a complex pathogenesis with an increasing prevalence and is one of the most intractable clinical challenges in the field of reproductive medicine. Quercetin (QCT) is an effective active ingredient extracted from Semen Cuscutae and Herba Taxilli used in traditional Chinese medicine for tonifyng the kidneys and promoting fetal restoration. Although QCT helps improve adverse pregnancy outcomes, the specific mechanism remains unclear. The trophoblast cell line HTR‐8/SVneo cultured in vitro was treated with different concentrations of QCT, and the cell counting kit‐8 assay, wound healing assay, transwell assay, and western blotting were used to evaluate the effects and mechanisms of QCT on the proliferation, migration, and invasion of HTR‐8/SVneo cells, respectively. To assess the expression levels of miR‐149‐3p and AKT serine/threonine kinase 1 (AKT1), quantitative real‐time polymerase chain reaction (qRT‐PCR) and western blotting analysis were performed. A dual‐luciferase reporter assay was used to investigate the potential regulatory relationship between miR‐149‐3p and AKT1. Our results showed that QCT promoted the proliferation, migration, and invasion of trophoblast cells, promoted the expression of MMP2, MMP9, and vimentin, and downregulated the expression of E‐cadherin. Mechanistically, QCT downregulated the expression of miR‐149‐3p and upregulated the expression of AKT1, and miR‐149‐3p directly targets AKT1, negatively regulating its expression. Overexpression of miR‐149‐3p and silencing of AKT1 counteracted the promotional effects of QCT on trophoblast proliferation, migration, and invasion. Taken together, QCT regulates the migration and invasion abilities of HTR‐8/SVneo cells through the miR‐149‐3p/AKT1 axis, which may provide a promising therapeutic approach for RSA.

## 1 INTRODUCTION

Recurrent spontaneous abortion (RSA) is typically defined as the occurrence of three or more spontaneous abortions before 20–28 weeks of gestation in women with consistent sexual partners. RSA is a major pregnancy complication affecting 1%–2% of women of childbearing age worldwide, and multiple recurrent abortions can lead to physical and mental health issues in affected women.^1^, ^2^ RSA involves a complex interplay of pathological processes that may be associated with the anchoring of trophoblast cells in the placenta during early pregnancy, remodeling of maternal spiral arteries, decidua angiogenesis, hormone and cytokine secretion, and crosstalk with maternal immune cells.^3^ Extrachorionic trophoblast cells play crucial roles in facilitating embryo implantation in the endometrium and remodeling of maternal spiral arteries to ensure adequate maternal blood supply for fetal growth and development.^4^ Placental dysfunction resulting from the reduced proliferation, migration, and invasive capacity of extrachorionic trophoblasts is implicated in RSA. Therefore, exploring the factors affecting trophoblast invasion is essential to enhancing our understanding of RSA pathogenesis.

MicroRNAs (miRNAs) are endogenous short, single‐stranded, noncoding RNAs, approximately 22 nucleotides in length, encoded by eukaryotes, that are involved in posttranscriptional regulation in plants and animals. They are crucial in various physiological and pathological processes such as cell differentiation, proliferation, apoptosis, angiogenesis, and inflammatory responses.^5^ Furthermore, miRNAs play critical roles in RSA pathogenesis. Liu et al. reported that miR‐93 expression was significantly elevated in the chorionic tissues of patients with RSA. Upregulation of miR‐93 inhibits the proliferation, migration, and invasive abilities of HTR‐8/SVneo cells and promotes apoptosis.^6^ Additionally, miR‐184 induces RSA development by promoting trophoblastic cell apoptosis by targeting WIG1.^7^ Patients with endometrial and ovarian cancers have elevated miR‐149‐3p expression. High miR‐149‐3p expression in patients with endometrial cancer was significantly associated with low survival rates.^8^ Similarly, in ovarian cancer, high miR‐149‐3p expression leads to resistance to cisplatin‐based drugs by targeting CDKN1A and TIMP2.^9^ In a related study by Tang et al., miR‐149‐3p was highly expressed in the placental villous tissues of patients with RSA.^10^ However, the role of miR‐149‐3p in regulating the migration and invasion of trophoblast cells remains unknown.

Traditional Chinese medicine (TCM) has gained considerable attention in reproductive medicine. Quercetin (QCT) is a naturally occurring flavonoid polyphenol with anti‐inflammatory, antibacterial, antioxidant, anticancer, and antiviral properties. Furthermore, QCT is an active ingredient found in TCMs such as Semen Cuscutae and Herba Taxilli, which are used as kidney tonics and to support fetal health. QCT can cross the placental barrier and affect the immune response at the maternal–fetal interface. This can improve the migration and invasion of extrachorionic trophoblasts, potentially leading to better pregnancy outcomes.^11^, ^12^ QCT improves HTR‐8/SVneo mitochondrial function under hypoxic conditions, increases trophoblast proliferation, and inhibits apoptosis by increasing miR‐34a‐5p expression and inhibiting Drp1.^13^ However, the exact mechanism through which QCT regulates trophoblast proliferation, migration, and invasion via miR‐149‐3p remains unknown. Therefore, this study explored the biological significance of the QCT/miR‐149‐3p/AKT serine/threonine kinase 1 (AKT1) axis in trophoblasts by culturing HTR‐8/SVneo cells in vitro. Our findings may provide a theoretical basis for the treatment of RSA with QCT.

## 2 MATERIALS AND METHODS

### 2.1 Patients and specimens

Patients admitted to the Second Affiliated Hospital of the Henan University of Chinese Medicine (Zhengzhou, China) between September 2022 and December 2023 were enrolled. Among them, 28 women with RSA and 18 healthy pregnant women were included in this study. The inclusion criteria were as follows: women with three or more consecutive pregnancy failures with the same partner before 20 weeks of gestation or those with unexplained RSA that cannot be attributed to known causes associated with RSA, such as karyotype abnormalities, uterine anatomy abnormalities, endocrine disorders, infectious pathology, or immune disorders, were included in the RSA group. Women with no specific medical reason, no history of abortion or other pregnancy complications, and who opted for voluntary termination of pregnancy were included as controls. Five milliliters of venous blood samples were collected from all patients before medical termination of pregnancy and placed in a coagulant tube. After incubation at room temperature, the sample was centrifuged at 3000 rpm for 10 min. The extracted serum was stored at −80°C in an Eppendorf tube void of RNA enzyme. The samples were collected after obtaining written informed consent from the participants. This study was approved by the Ethics Committee of the Second Affiliated Hospital of the Henan University of Chinese Medicine.

### 2.2 Cell culture

The human trophoblast cell line HTR‐8/SVneo was obtained from the China Center for Type Culture Collection (Wuhan, China). The cells were cultured in 1640 medium with 10% fetal bovine serum (FBS; Hyclone, South Logan, USA), 100 U/mL penicillin, and 100 μg/mL streptomycin (Sangon Biotech, Shanghai, China) in a humidified atmosphere containing 5% CO_2_ and 95% humidity at 37°C.

### 2.3 Preparation of QCT for cell treatment

Powdered QCT (Cas No. 117‐39‐5, Beijing Solarbio Science, China) was dissolved in dimethyl sulfoxide (DMSO) to achieve a 10 mM stock solution and stored at −20°C. QCT was further diluted with RPMI 1640 medium to achieve final concentrations of 1, 2, and 5 μM. When the cell density reached approximately 90%, the 2% FBS 1640 medium was replaced with fresh medium for 12 h. Subsequently, the QCT‐treated group received medium supplemented with 1, 2, and 5 μM QCT containing 10% FBS. The control group received the same amount of medium without QCT.

### 2.4 Plasmid construction and transfection

Negative control (NC), miR‐149‐3p mimic, and miR‐149‐3p inhibitor were acquired from RiboBio (Shanghai, China), and pEX‐AKT1 and si‐AKT1 were obtained from GenePharma (Shanghai, China). HTR‐8/SVneo cells in healthy and logarithmic growth phases were seeded into a 6‐well plate at a density of 2 × 10^5^ cells/mL. The cells were allowed to incubate overnight until they reached 80% confluence. Following the manufacturer's instructions, the cells were transfected with NC, miR‐149‐3p mimics, miR‐149‐3p inhibitors, pEX‐AKT1, or si‐AKT1 using Lipofectamine 3000 (Invitrogen, Carlsbad, CA, USA) for 24 h prior to conducting other assays.

### 2.5 RNA extraction and qRT‐PCR

Total RNA was extracted from the cells using TRIzol reagent (Sangon Biotech, Shanghai, China) following the manufacturer's instructions. The concentration and purity of the total RNA were determined using a spectrophotometer (NanoDrop 2000c, Thermo Fisher Scientific, USA). The miRNA‐specific reverse primers were used for cDNA synthesis. Real‐time PCR was performed using SYBR Green Fast qPCR Master Mix (Sangon Biotech, Shanghai, China) on a 7500 Detection System, according to the manufacturer's guidelines. The expression levels of miR‐149‐3p were normalized to those of the small nuclear RNA U6, and GAPDH was used as an endogenous control for AKT1. The relative gene expression level was calculated using the 2^−ΔΔCt^ method. The primer sequences used in this study are listed in Table 1.

**TABLE 1:** Primer sequences.

Primers	Sequences (5′–3′)
U6‐F	CTCGCTTCGGCAGCACA
U6‐R	AACGCTTCACGAATTTGCGT
miR‐149‐3p RT	GTCGTATCCAGTGCAGGGTCCGAGGTATTCGCACTGGATACGACGCACAG
miR‐149‐3p‐F	ACAGGGAGGGACGGGGG
miR‐149‐3p‐R	CAGTGCAGGGTCCGAGGTATT
GAPDH‐F	CACCCACTCCTCCACCTTTGAC
GAPDH‐R	GTCCACCACCCTGTTGCTGTAG
AKT1‐F	ACTGTCATCGAACGCACCTTCC
AKT1‐R	TCTCCTCCTCCTCCTGCTTCTTG

### 2.6 Western blotting

Total proteins from cells were extracted by adding RIPA lysis buffer (Solarbio Science, Beijing, China) containing phenylmethylsulfonyl fluoride at 4°C. The following antibodies were used for western blotting analysis: AKT1 (dilution 1:1000, catalog number HY‐P74421, MedChem Express), MMP2 (dilution 1:800, catalog number 10373‐2‐AP, Proteintech Group), MMP9 (dilution 1:1000, catalog number 10375‐2‐AP, Proteintech Group), Vimentin (dilution 1:20000, catalog number ET1610‐39, Huabio), E‐cadherin (dilution 1:1000, catalog number ET1607‐75, Huabio), and GAPDH (dilution 1:1000; catalog number GB15004, Servicebio). The antibodies were diluted according to the standard protocols. The same amount of protein cell lysate was separated through sodium dodecyl sulfate‐polyacrylamide gel electrophoresis, transferred onto a polyvinylidene fluoride membrane (Millipore, USA), and subsequently blocked with 5% skimmed milk for 2 h at 25°C. The membranes were then incubated with primary antibodies overnight at 4°C, followed by incubation with a secondary antibody, specifically a horse radish peroxidase‐conjugated anti‐rabbit IgG (dilution 1:5000, catalog number GB23303, Servicebio), for 1 h at 37°C. The protein bands were quantified using an electrochemiluminescence reagent (Millipore Sigma, USA) and analyzed using ImageJ software (National Institutes of Health, USA). GAPDH was used as a control for the normalization of total protein.

### 2.7 Dual‐luciferase reporter assay

The predicted AKT1 wild type (WT) or mutant (MUT) 3′‐untranslated region (UTR) containing the miR‐149‐3p binding site was inserted into the luciferase reporter gene vector (RiboBio, Shanghai, China) to construct the AKT1‐WT and AKT1‐MUT plasmids. The WT or MUT AKT1 3′‐UTR plasmid and miR‐149‐3p mimics or mimic‐NC were co‐transfected into HEK‐293 T cells using Lipofectamine 3000 transfection reagent. After 48 h, luciferase activity was determined using a dual‐luciferase reporter assay system (Promega, Madison, USA). Renilla luciferase activity was used to normalize firefly luciferase activity.

### 2.8 Cell counting kit‐8 (CCK8) assay

The cells were seeded into 96‐well plates at a density of 5 × 10^4^ cells/mL and incubated for 0, 24, 48, or 72 h. Cells were then treated with 10 μL of CCK‐8 solution (Servicebio, Wuhan, China) at 37°C for 2 h after the incubation period. The absorbance was measured at 450 nm using a microplate reader.

### 2.9 Wound healing assay

When the cells reached 90% confluence, the cell monolayer was scratched vertically using a sterilized 100 μL pipette. Medium containing 1% FBS was then added, and the wound healing area was observed by imaging the cells using a phase‐inverted microscope (Nikon, Tokyo, Japan) at 100× magnification immediately and after for 0 h and 24 h. The average percentage of wound closure was calculated using ImageJ software using the formula: wound healing percent = [(*A* − *B*)/*A*] × 100%, where *A* and *B* denote the initial and remaining wound areas at 0 h and 24 h after cell migration, respectively.

### 2.10 Transwell migration and invasion assays

Transwell assays were used to observe the migration and invasion abilities of HTR‐8/SVneo cells. Transwell inserts with 8‐μm pore size membranes (Corning, NY, USA) were used. In migration assessments, 1 × 10^5^ cells per well were resuspended in 100 μL serum‐free RPMI 1640 medium in the upper transwell insert chamber, and 600 μL of RPMI 1640 medium containing 10% FBS was added to the lower chambers. After 24 h, cells were fixed with 4% paraformaldehyde at 20°C for 30 min and stained with 0.1% crystal violet at 20°C for 10 min. The number of cells migrating to the underside was counted using a light microscope (100× magnification) in five random visual fields per plate. Invasion assays were performed in an upper transwell insert chamber precoated with Matrigel (BD Biosciences, San Jose, USA) and incubated at 37°C for 3 h. Other procedures were the same as those used for migration assays.

### 2.11 Annexin V/FITC‐PI apoptosis assays

Following the manufacturer's instructions for apoptosis detection reagent, the cells were digested with trypsin without ethylene diamine tetra‐acetic acid (Sangon Biotech, Shanghai, China), and the cell supernatant was collected simultaneously and centrifuged at 4°C, 500 *g*, for 5 min. The cells were resuspended in 1× binding buffer at a density of 5 × 10^5^ cells/mL and incubated with Annexin V‐FITC and PI for 8 min at room temperature in the dark (Servicebio, Wuhan, China). Within 1 h, the apoptotic cells were assessed using flow cytometry (FACScan, BD Biosciences, USA).

### 2.12 Statistical analysis

All data were analyzed using GraphPad Prism 8 (La Jolla, CA, USA); images were analyzed using ImageJ, and image combinations were analyzed using Adobe Illustrator. The data are presented as the mean ± standard deviation (SD) of at least three independent analyses. Student's *t*‐test was used to examine the differences between the two independent groups, and one‐way analysis of variance by Tukey's post hoc test was used to assess the differences between more than two groups. Diagnostic utility was measured using the receiver operating characteristic (ROC) curve analysis. A *p*‐value <0.05 indicates statistical significance.

## 3 RESULTS

### 3.1 QCT promotes proliferation, migration, and invasion and inhibits apoptosis in HTR‐8/SVneo cells

**FIGURE 1:**
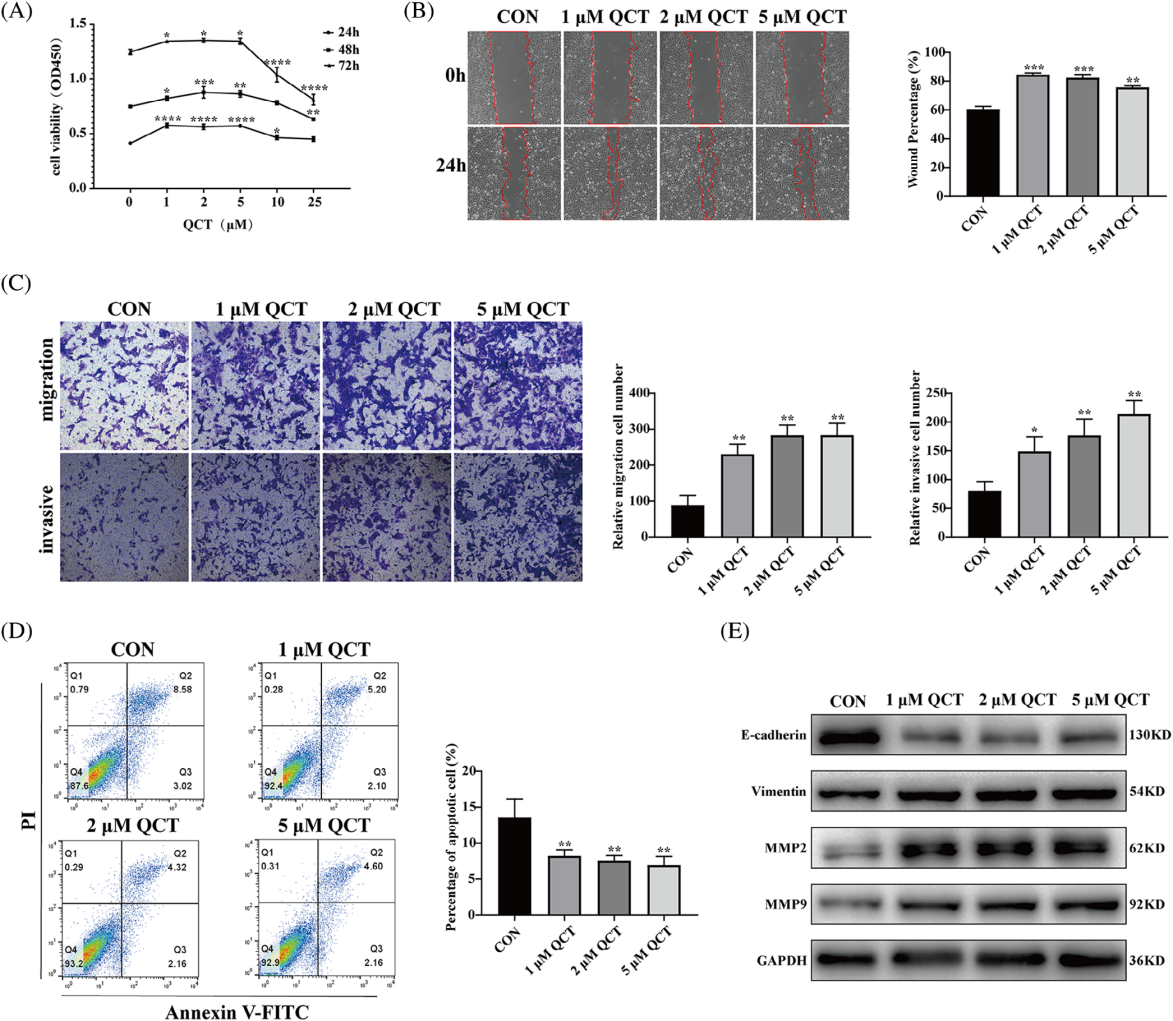
Quercetin (QCT) promotes proliferation, migration, and invasion, and inhibits apoptosis in HTR‐8/SVneo cells. (A) Effect of 1, 2, 5, 10, and 25 μM QCT on cell viability after treatment of trophoblast cells at 24, 48, and 72 h was detected by CCK8. (B) Effects of 1, 2, and 5 μM QCT on cell migration were determined using a wound healing assay. (C) Effect of 1, 2, and 5 μM QCT on cell migration and invasion were detected using the transwell assay. Magnification, ×100. (D) Effect of 1, 2, and 5 μM QCT on apoptosis were detected by flow cytometry. (E) Western blotting detection of MMP2, MMP9, vimentin, and E‐cadherin in HTR‐8/SVneo cells treated with 1, 2, and 5 μM QCT. Data are presented as the mean ± standard deviation (SD). *p < 0.05; **p < 0.01; ***p < 0.001; ****p < 0.0001. CCK8, cell counting kit‐8.

To determine whether QCT mitigates RSA progression, different concentrations of 0, 1, 5, 10, and 25 μM QCT were administered to HTR‐8/SVneo cells for 24, 48, and 72 h, and cell viability was determined using CCK‐8. Our results showed that QCT at 1, 2, and 5 μM significantly promoted cell viability at 24, 48, and 72 h, whereas 10 and 25 μM downregulated cell viability. Based on these results, HTR‐8/SVneo cells were treated with 1, 2, and 5 μM QCT in subsequent experiments (Figure 1A). Wound healing assay results showed that 1, 2, and 5 μM QCT significantly promoted cell migration (*p* < 0.01, Figure 1B). In addition, the results of the transwell assay indicated that QCT promoted the invasion of HTR‐8/SVneo cells (*p* < 0.05, Figure 1C). The apoptosis assay results showed that QCT significantly attenuated the apoptotic ability of HTR‐8/SVneo cells (*p* < 0.01, Figure 1D). Western blotting analysis was then performed to analyze the expression of the markers MMP2, MMP9, E‐cadherin, and vimentin, which are associated with migration and invasion in HTR‐8/SVneo cells after QCT treatment. The results showed that, compared with the control group, QCT at concentrations of 1, 2, and 5 μM promoted the expression of MMP2, MMP9, and vimentin and inhibited the expression of E‐cadherin (Figure 1E). These findings suggest that QCT plays an important role in trophoblast cell development and embryo implantation by regulating cell migration and invasion.

### 3.2 QCT downregulates miR‐149‐3p

**FIGURE 2:**
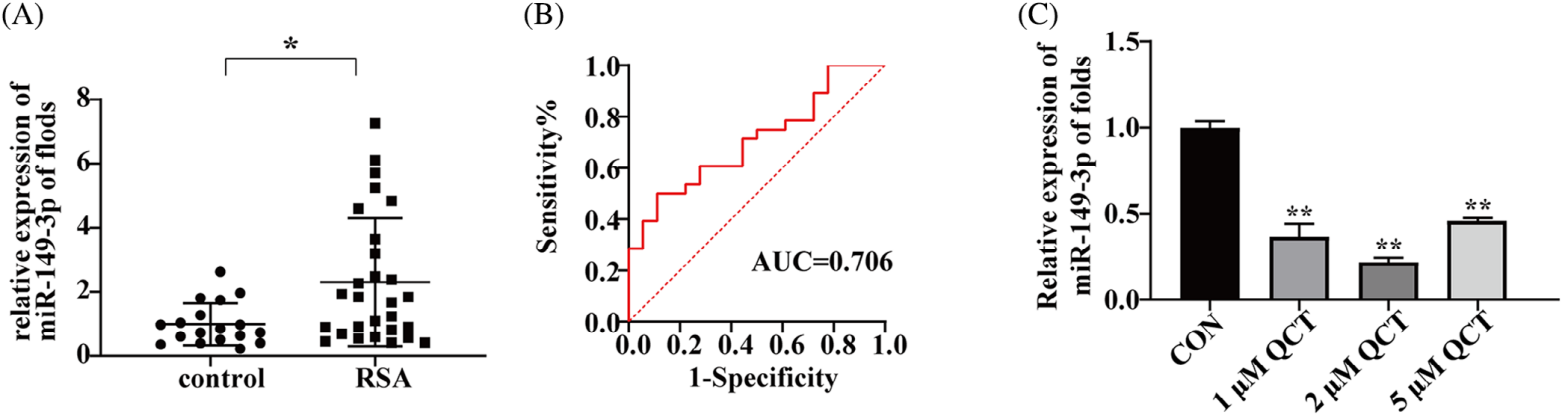
QCT downregulates miR‐149‐3p. (A) Expression of miR‐149‐3p in the serum of patients in the recurrent spontaneous abortion (RSA) (*n* = 28) and control groups (*n* = 18) was detected using quantitative real‐time polymerase chain reaction (qRT‐PCR). (B) ROC curve of serum miR‐149‐3p. (C) qRT‐PCR detected miR‐149‐3p levels in HTR‐8/SVneo cells after 1, 2, and 5 μM QCT treatment. Relative gene expression was normalized to that of U6. Data are presented as the mean ± SD. The results are reported as fold changes compared with the control group. *p < 0.05; **p < 0.01. QCT, quercetin; ROC, receiver operating characteristic.

The qRT‐PCR assay was performed to detect the expression of miR‐149‐3p in the serum of 28 patients in the RSA group and 18 patients in the control group, which revealed that the serum expression levels of miR‐149‐3p were significantly higher in the RSA group than in the control group (*p* < 0.05, Figure 2A). The results of the ROC curve analysis showed that the sensitivity and specificity of miR‐149‐3p were 50% and 88.9%, respectively, with an AUC of 0.706 (95% CI: 0.558–0.855, *p* < 0.05), and this difference was statistically significant (Figure 2B). Interestingly, QCT treatment downregulated miR‐149‐3p expression (*p* < 0.01, Figure 2C). In conclusion, QCT may affect the biological function of trophoblast by regulating miR‐149‐3p.

### 3.3 miR‐149‐3p inhibits proliferation, migration, and invasion and promotes apoptosis in HTR‐8/SVneo cells

**FIGURE 3:**
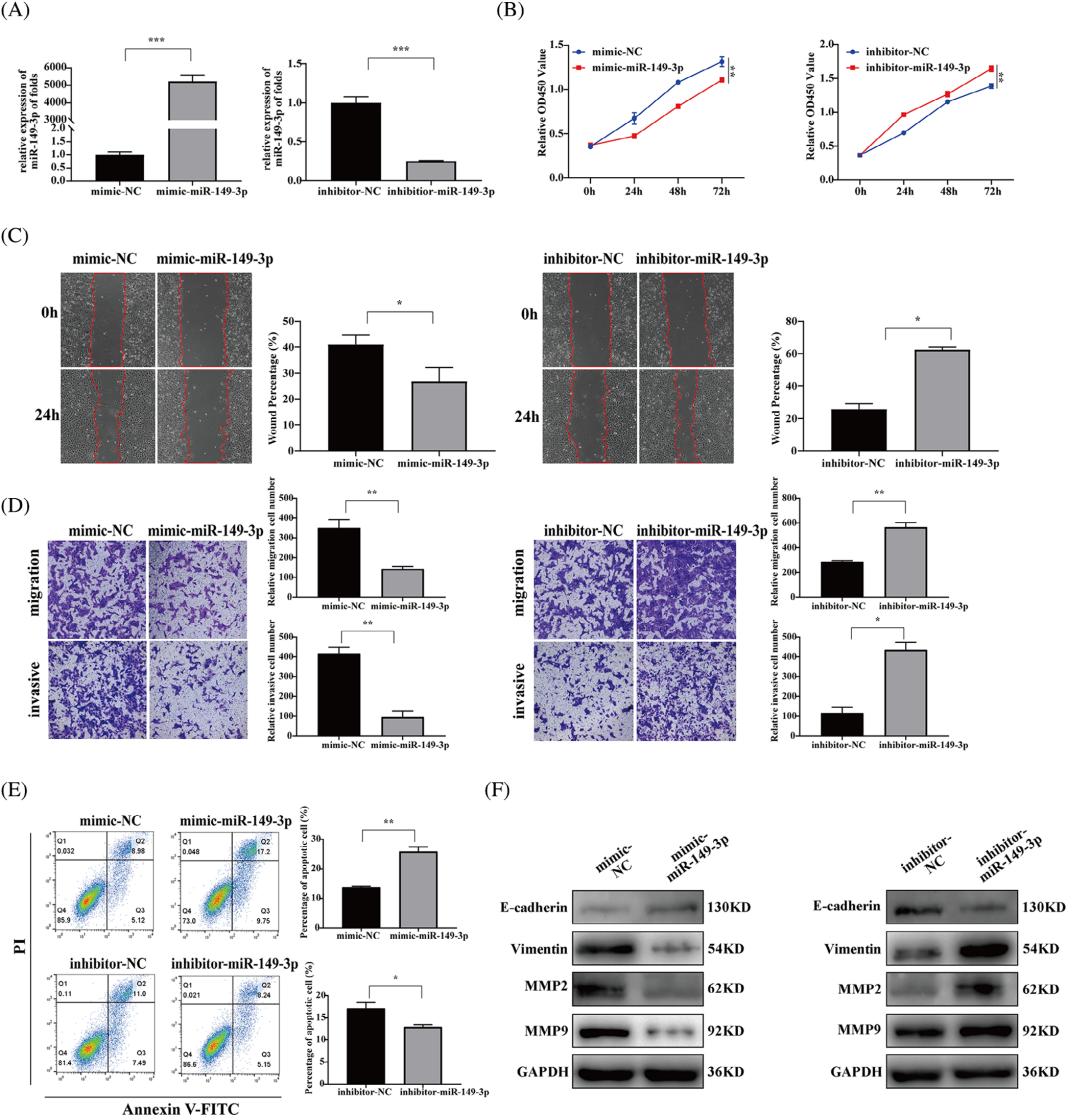
miR‐149‐3p inhibits proliferation, migration, and invasion and promotes apoptosis in HTR‐8/SVneo cells. (A) qRT‐PCR detected the relative expression levels of miR‐149‐3p in HTR‐8/SVneo cells after transfection with miR‐149‐3p mimic and inhibitor. (B) CCK8 was used to determine the proliferative capacity of mimic‐miR‐149‐3p and inhibitor‐miR‐149‐3p transfected HTR‐8/SVneo cells at 0, 24, 48 and 72 h. (C) Wound healing assay and quantitation were performed to examine migration ability after transfection with mimic‐miR‐149‐3p or inhibitor‐miR‐149‐3p for 24 h. (D) Transwell assays and quantitation were performed to assess the migration and invasiveness of HTR‐8/SVneo cells. Magnification, 100×. (E) Apoptosis of HTR‐8/SVneo cells was determined by flow cytometry. (F) Effects of mimic‐miR‐149‐3p and inhibitor‐miR‐149‐3p on MMP2, MMP9, vimentin, and E‐cadherin protein expression levels detected by western blotting. Data are presented as the mean ± SD. *p < 0.05; **p < 0.01; ***p < 0.001. CCK8, cell counting kit‐8; qRT‐PCR, quantitative real‐time polymerase chain reaction.

For functional experiments, mimic‐NC, mimic‐miR‐149‐3p, inhibitor‐NC, and inhibitor‐miR‐149‐3p were transfected into HTR‐8/SVneo cells using liposome technology. A qRT‐PCR assay was performed to determine transfection efficiency, which showed that miR‐149‐3p overexpression and silencing were achieved in HTR‐8/SVneo cells. (*p* < 0.01, Figure 3A). The results of the CCK8 assay showed that, mimic‐miR‐149‐3p significantly inhibited the proliferation of HTR‐8/SVneo cells than did mimic‐NC, whereas inhibitor‐miR‐149‐3p showed significantly higher viability of HTR‐8/SVneo cells than did inhibitor‐NC (*p* < 0.01, Figure 3B). In addition, mimic‐miR‐149‐3p significantly inhibited HTR‐8/SVneo cell migration and invasion than did mimic‐NC, whereas inhibitor‐miR‐149‐3p had a significantly higher capacity for HTR‐8/SVneo cell migration and invasion than did inhibitor‐NC (Figure 3C,D). The apoptosis assay showed that the apoptosis rate in HTR‐8/SVneo cells treated with mimic‐miR‐149‐3p was significantly higher than in those treated with mimic‐NC. Moreover, the apoptosis rate in HTR‐8/SVneo cells treated with inhibitor‐miR‐149‐3p was lower than in those treated with inhibitor‐NC (Figure 3E). Western blotting results showed that mimic‐miR‐149‐3p inhibited the expression of MMP2, MMP9, and vimentin and promoted the expression of E‐cadherin compared to mimic‐NC, whereas miR‐149‐3p silencing appeared to have the opposite effect (Figure 3F).

### 3.4 QCT promotes proliferation, migration, and invasion of HTR‐8/SVneo cells via miR‐149‐3p

**FIGURE 4:**
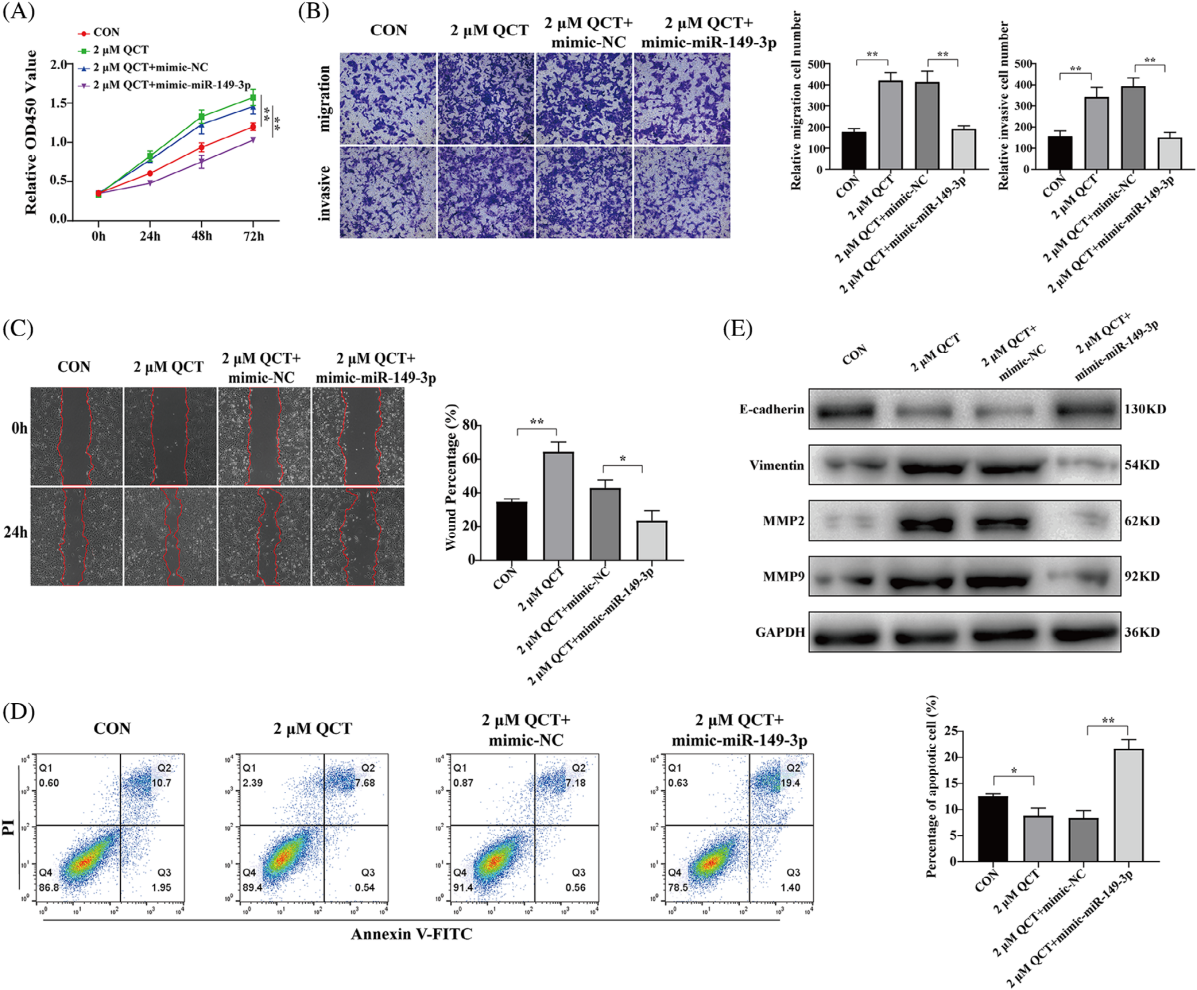
QCT promotes proliferation, migration, and invasion of HTR‐8/SVneo cells via miR‐149‐3p. (A–D) CCK8, transwell, wound healing, and apoptosis assays were used to assess the effects of miR‐149‐3p overexpression on proliferation, migration, invasion, and apoptosis of QCT‐treated HTR‐8/SVneo cells. (E) Protein expression levels of MMP2, MMP9, vimentin, and E‐cadherin in different treatment groups as determined by western blotting. Data are presented as the mean ± SD. *p < 0.05; **p < 0.01. CCK8, cell counting kit‐8; QCT, quercetin.

To determine whether QCT regulated RSA through miR‐149‐3p, rescue assays using mimic‐miR‐149‐3p were conducted. CCK8, transwell, wound healing, and apoptosis assays showed that mimic‐miR‐149‐3p significantly attenuated the effects of QCT on the proliferation, migration, and invasion of HTR‐8/SVneo cells and promoted apoptosis (Figure 4A–D). Western blotting results showed that QCT promoted the expression of MMP2, MMP9, and vimentin and inhibited the expression of E‐cadherin; these effects were reversed by miR‐149‐3p overexpression (Figure 4E).

### 3.5 miR‐149‐3p directly targets AKT1

**FIGURE 5:**
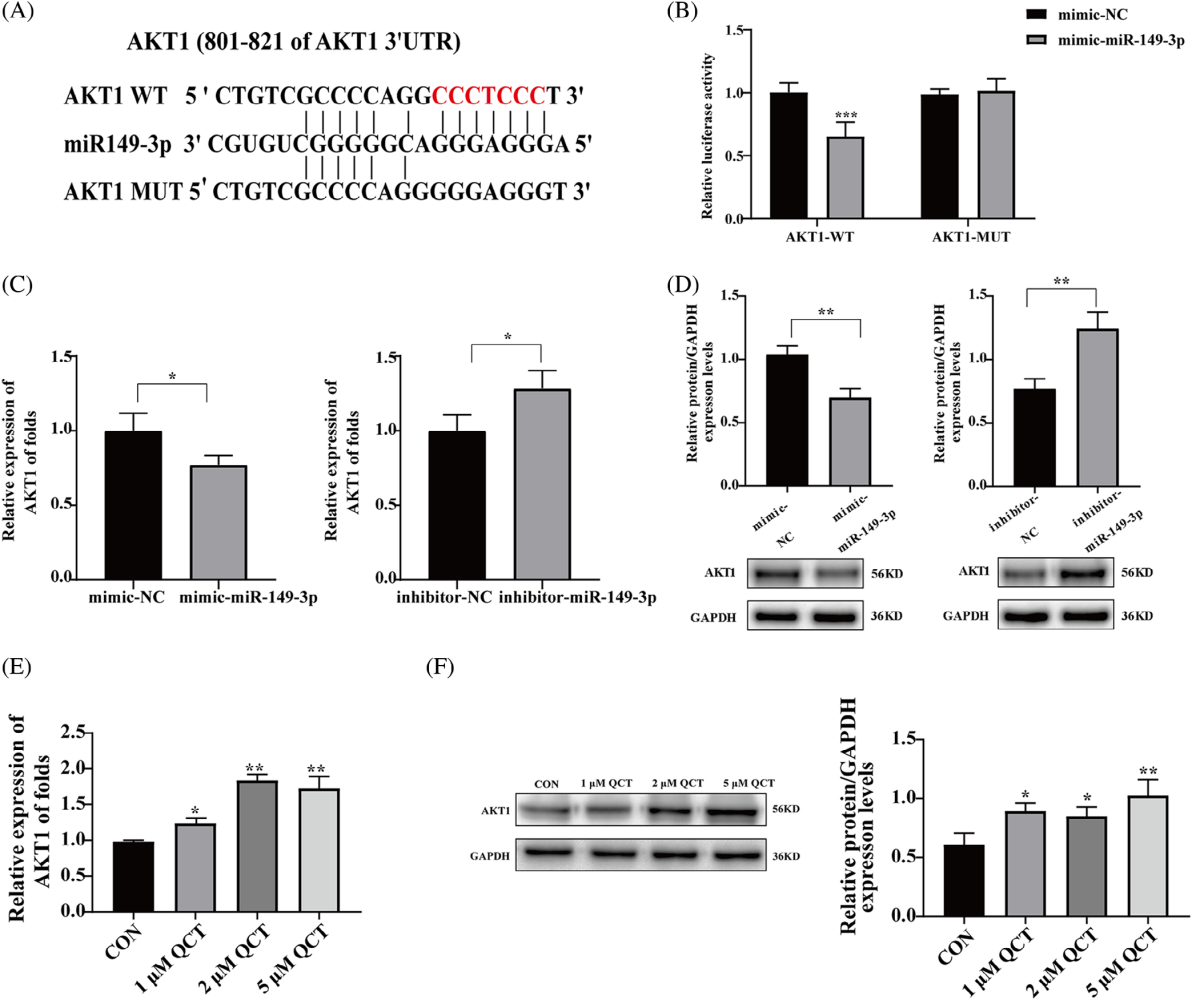
miR‐149‐3p directly targets AKT serine/threonine kinase 1 (AKT1). (A) Predicted miR‐149‐3p binding target site in the AKT1 mRNA 3′‐ untranslated region (UTR) in the TargetScan, miRDB, and miRWalk databases. (B) Wild type (WT) or mutant (Mut) miR‐149‐3p binding sites in the AKT1 3′‐UTR reporter genes and miR‐149‐3p mimics or inhibitors were transfected into HEK‐293 T cells, and the fluorescence intensity was measured using a luciferase detection kit after 24 h. (C) HTR‐8/SVneo cells were transfected with mimic‐149‐3p or inhibitor‐miR‐149‐3p. qRT‐PCR was used to detect AKT1 mRNA expression levels. (D) Western blotting detection of AKT1 protein expression levels after mimic‐149‐3p or inhibitor‐miR‐149‐3p transfection. (E) Effect of 1, 2, and 5 μM QCT treatment on the expression level of AKT1 mRNA in HTR‐8/SVneo cells detected by qRT‐PCR. (F) Western blotting detection of the effect of 1, 2, and 5 μM QCT treatment on AKT1 protein expression levels in HTR‐8/SVneo cells. ImageJ software was used to analyze the gray scale values of each group and calculate the relative expression of proteins. Data are presented as the mean ± SD. *p < 0.05; **p < 0.01; ***p < 0.001. QCT, quercetin; qRT‐PCR, quantitative real‐time polymerase chain reaction.

Three bioinformatics software, TargetScan (https://www.targetscan.org/vert_80/), miRDB (http://mirwalk.umm.uni-heidelberg.de/), and miRWalk (http://mirwalk.umm.uni-heidelberg.de/) were used to determine the target genes for miR‐149‐3p. The database results showed that AKT1 was a potential target gene of miR‐149‐3p (Figure 5A). To verify the binding between miR‐149‐3p and AKT1 mRNA, the AKT1 WT/AKT1 MUT‐type dual‐luciferase reporter plasmid and mimic‐NC/mimic‐miR‐149‐3p were transfected into HEK‐293 T cells. The results showed that miR‐149‐3p overexpression significantly downregulated the luciferase activity of AKT1‐WT than did mimic‐NC (*p* < 0.001, Figure 5B). However, mimic‐NC and mimic‐miR‐149‐3p did not affect the luciferase activity of AKT1‐MUT (*p* > 0.05, Figure 5B). Furthermore, qRT‐PCR and western blotting results revealed that overexpression of miR‐149‐3p decreased AKT1 mRNA and protein expression. Conversely, silencing miR‐149‐3p increased AKT1 mRNA and protein expression (Figure 5C,D). The results of qRT‐PCR and western blotting showed that treatment with 1, 2, and 5 μM of QCT resulted in a significant upregulation of the expression of AKT1 mRNA and protein in HTR‐8/SVneo cells (Figure 5E,F). These data suggest that AKT1 is targeted by miR‐149‐3p.

### 3.6 Upregulation of AKT1 counteracts the effects of miR‐149‐3p on proliferation, migration, and invasion of HTR‐8/SVneo cells

**FIGURE 6:**
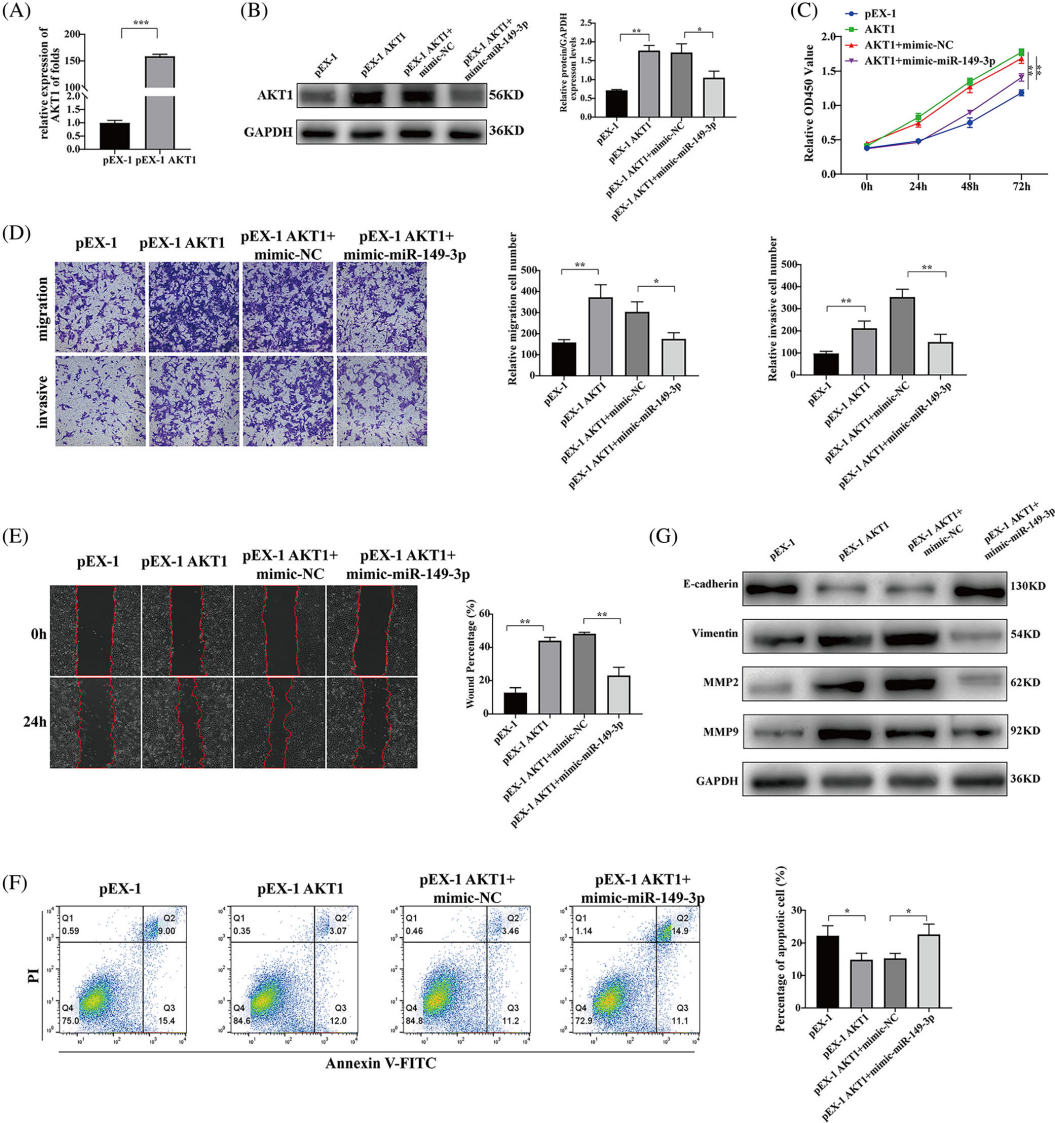
Upregulation of AKT1 counteracts the effects of miR‐149‐3p on proliferation, migration, and invasion of HTR‐8/SVneo cells. (A and B) Detection of AKT1 mRNA and protein expression levels in HTR‐8/SVneo cells by RT‐PCR and western blotting. (C–F) CCK8, transwell, wound healing, and apoptosis assays for the effects of miR‐149‐3p on proliferation, migration, invasion, and apoptosis of HTR‐8/SVneo cells overexpressing AKT1. (G) Effect of overexpression of miR‐149‐3p on the expression levels of MMP2, MMP9, vimentin, and E‐cadherin proteins in HTR‐8/SVneo cells overexpressing AKT1 detected by western blotting. Data are presented as the mean ± SD. *p < 0.05; **p < 0.01; ***p < 0.001. AKT1, AKT serine/threonine kinase 1; RT‐PCR, real‐time polymerase chain reaction.

The pEX‐1 and pEX‐1 AKT1 plasmids were transfected into HTR‐8/SVneo cells for 48 h, and the expression level of AKT1 was assessed using qRT‐PCR (*p* < 0.001, Figure 6A). Subsequently, rescue experiments were performed to examine whether miR‐149‐3p directly targets AKT1 to inhibit the viability of HTR‐8/SVneo cells. The western blotting results showed that pEX‐1 AKT1 overexpression was able to promote AKT1 expression compared to the pEX‐1 group. In contrast, the presence of miR‐149‐3p mimic inhibited AKT1 expression (*p* < 0.01; Figure 6B). The CCK8 assay results showed that the pEX‐1 AKT1 overexpression group had higher cell viability than that of the pEX‐1 group (*p* < 0.01). Furthermore, the pEX‐1 AKT1 + miR‐149‐3p group inhibited cell viability by miR‐149‐3p overexpression (*p* < 0.01; Figure 6C). The wound healing assay and transwell assay results showed that cell migration and invasion was significantly higher in the pEX‐1 AKT1 overexpression group than in the pEX‐1 group (*p* < 0.01). The promotion of cell migration and invasion by pEX‐1 AKT1 overexpression was significantly reversed by mimic‐miR‐149‐3p (Figure 6D,E). Apoptosis assays showed that, compared with the pEX‐1 group, the pEX‐1 AKT1 overexpression group demonstrated significant inhibition of apoptosis (*p* < 0.01). In contrast, co‐transfection of mimic‐miR‐149‐3p and pEX‐1 AKT1 significantly increased apoptosis rates in HTR‐8/SVneo cells (*p* < 0.05; Figure 6F). Western blotting results showed that AKT1 overexpression promoted the expression of proteins related to migration and invasion in HTR‐8/SVneo cells, whereas overexpression of miR‐149‐3p reversed this effect (Figure 6G). These findings indicated that miR‐149‐3p inhibits cell proliferation and promotes apoptosis by downregulating AKT1 expression in HTR‐8/SVneo cells.

### 3.7 QCT promotes proliferation, migration, and invasion of HTR‐8/SVneo cells via AKT1

**FIGURE 7:**
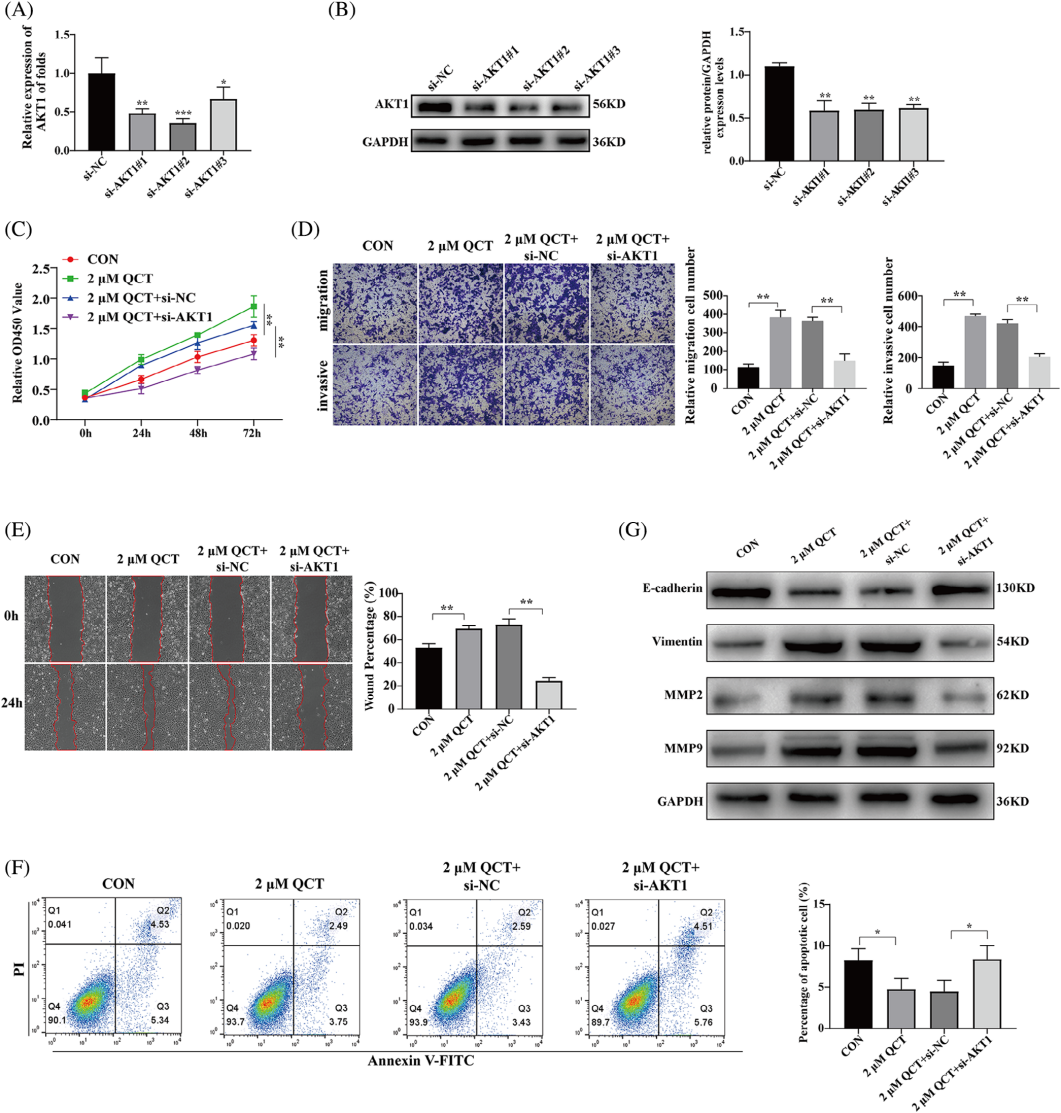
QCT promotes proliferation, migration, and invasion of HTR‐8/SVneo cells via AKT1. (A and B) Knockdown efficiency of si‐AKT1 detected using qRT‐PCR and western blotting. (C–F) CCK8, transwell, wound healing, and apoptosis assays to assess the effects of AKT1 knockdown on proliferation, migration, invasion, and apoptosis of QCT‐treated HTR‐8/SVneo cells. (G) Western blotting evaluation of the effect of AKT1 knockdown on MMP2, MMP9, vimentin, and E‐cadherin protein expression levels in QCT‐treated HTR‐8/SVneo cells. Data are presented as the mean ± SD. *p < 0.05; **p < 0.01; ***p < 0.001. AKT1, AKT serine/threonine kinase 1; QCT, quercetin; qRT‐PCR, quantitative real‐time polymerase chain reaction.

To determine whether QCT regulates RSA via the miR‐149‐3p/AKT1 axis, rescue assays using si‐AKT1 were performed. The qRT‐PCR and western blotting results showed that AKT1 was significantly knocked down in HTR‐8/SVneo cells (Figure 7A,B). CCK8, transwell, wound healing, and apoptosis assays showed that QCT promoted cell proliferation, migration, and invasion and inhibited apoptosis, and AKT1 knockdown partially abrogated the protective effect of QCT on cells (Figure 7C–F). AKT1 knockdown reversed QCT on the expression of proteins associated with migration and invasion in HTR‐8/SVneo cells (Figure 7G). These data suggest that QCT promotes HTR‐8/SVneo cell migration, and invasion is partly mediated through the miR‐149‐3p/AKT1 axis.

## 4 DISCUSSION

RSA is the most common pregnancy‐related complication in women of childbearing age, and insufficient trophoblast invasion is strongly associated with impaired embryo implantation and placenta formation. Ubiquitin‐specific protease 25, a gene associated with the regulation of invasion and migration, is downregulated in the placental villi of patients with RSA, and its low expression inhibits the migration and invasion of trophoblast cells, which in turn affects embryo implantation and development.^14^ Luan et al. found that inactivation of CCL19 and CCR7 inhibits the downstream AKT pathway, which in turn downregulates MMP2 expression and affects trophoblast invasion.^15^ Although FPR2 expression inhibits trophoblast migration and invasion, its knockdown results in significantly higher levels of trophoblast invasion, migration, and angiogenesis, which promote embryo implantation.^16^ However, the mechanisms underlying trophoblast dysfunction in patients with RSA remain unclear. This study explored the pathogenic mechanisms affecting trophoblast migration and invasion to determine their impact on embryo implantation and placental development.

Owing to its low toxicity and cost, QCT has a wide range of clinical applications as a monomeric component in TCM. QCT has a broad spectrum of potential therapeutic applications, including neurodegenerative diseases, diabetes, obesity, and tumors. TCM has demonstrated potential therapeutic effects in preventing and treating adverse pregnancy outcomes, which not only improves the pregnancy rate but also reduces side effects such as unstable efficacy, concurrent infections, and allergies that occur with Western medicine treatments.^17^, ^18^ The ameliorative and therapeutic effects of QCT on RSA have been demonstrated previously. An animal study showed that the combined use of QCT and bromelain acetate during the first trimester of pregnancy in mice reduced the CD4/CD8 ratio and IFN‐γ/IL‐4 ratio and shifted the Th1/Th2 balance, thereby promoting pregnancy onset and increasing offspring survival.^19^ Furthermore, the antioxidant properties of QCT inhibit reactive oxygen species (ROS) production, modulate mitochondrial function, promote trophoblast fusion, and reduce the incidence of pregnancy‐related complications due to placental dysplasia.^20^ Our findings elucidated that QCT at concentrations of 1, 2, and 5 μM promoted the proliferation, migration, and invasion of HTR‐8/SVneo cells and inhibited apoptosis without a dose‐dependent effect. This suggests the potential therapeutic efficacy of QCT for treating RSA.

The miRNAs have gained attention in recent research for their roles in regulating trophoblast migration and invasion and may serve as biomarkers for diagnosing RSA. For example, Gu et al. showed that miR‐3074‐5p promoted RSA by inhibiting trophoblast invasion and inducing apoptosis.^21^ Another study found that M1‐type macrophage outer membrane vesicles could deliver miR‐146a‐5p and miR‐146b‐5p to trophoblast cells, which in turn inhibited trophoblast cell migration and invasion, leading to the negative regulation of epithelial‐mesenchymal transition in trophoblast cells.^22^ Compared with healthy controls, miR‐127‐3p is expressed at low levels in placental villi and serum samples from patients with RSA, and overexpression of miR‐127‐3p results in a significant increase in the invasive ability of trophoblast cells. In contrast, miR‐486‐5p was weakly expressed in the placental villi and plasma samples, and the diagnostic sensitivity and specificity of the miR‐127a‐3p and miR‐486‐5p combination were 88.9% and 80.0%, respectively.^23^ The miRNA‐149‐3p, which is involved in disease initiation and progression through the regulation of cell proliferation, apoptosis, cell cycle, and metastasis has been extensively investigated.^24^ For example, miR‐149‐3p reduces the viability and proliferative capacity of oral squamous cell carcinoma by decreasing AKT2 expression and increasing its sensitivity to the chemotherapeutic agent 5‐fluorouracil.^25^ In bladder cancer, increased miR‐149‐3p expression significantly inhibited proliferation, migration, and invasion.^26^ Moreover, miR‐149‐3p induces apoptosis and inhibits cell growth in gastric cancer, thereby exerting an antitumor effect.^27^ In our study, miR‐149‐3p was upregulated in the serum of patients with RSA, and ROC curve analysis of miR‐149‐3p could sensitively discriminate patients with RSA from healthy controls. In vitro experiments showed that upregulation of miR‐149‐3p decreased the proliferation, migration, and invasion of trophoblast cells and promoted apoptosis. The opposite result was observed after miR‐149‐3p inhibition, suggesting that miR‐149‐3p plays a critical role in the invasion of trophoblast cells into the myometrium. Notably, rescue assays validated that miR‐149‐3p is a downstream molecule that reverses the promoting effects of QCT on cell proliferation, migration, and invasion of HTR‐8/SVneo.

AKT1, also known as protein kinase B (PKB), is an oncogenic kinase that is overexpressed and highly phosphorylated in most cancers. AKT1 is involved in multiple signaling pathways that regulate cell survival, growth, and proliferation, and is considered an essential target for cancer therapy.^28^ AKT1 is widely expressed in the placental trophoblasts during early pregnancy and plays a crucial role in promoting placental development and function. AKT1‐deficient pregnant mice had marked placental dystrophy, reduced basement membranes, loss of saccharide‐containing cells in spongy trophoblasts, and reduced blood vessel formation, leading to placental insufficiency and impaired fetal development.^29^, ^30^ Kozai et al. showed that AKT1^−/−^ mice have a reduced ability of extravillous trophoblast cells to invade the uterus by inhibiting the phosphorylation of their downstream FOSO4, which in turn impairs embryonic and postnatal fetal growth.^31^ In this study, miR‐149‐3p was predicted to bind to the 3′‐UTR of AKT1 by a biological database during the early stage of the study. Using a dual‐luciferase reporter gene assay, we found that miR‐149‐3p directly targeted the 3′‐UTR of AKT1. These results were consistent with the results of another study on human adipose‐derived stem cells (hADSCs), in which miR‐149‐3p targeted AKT1 to promote osteogenic differentiation of hADSCs.^32^ Furthermore, miR‐149 inhibited AKT1 expression and reduced the proliferative capacity of the glioblastoma multiforme U87MG cell line.^33^ Our results showed that QCT upregulates AKT1 expression. Rescue experiments were performed to confirm that the miR‐149‐3p/AKT1 axis mediated the promotional effect of QCT on the proliferation, migration, and invasion of HTR‐8/SVneo cells.

Furthermore, our findings revealed that QCT at 10 and 25 μM decreased the viability of HTR‐8/SVneo cells and had a more pronounced effect at 72 h. The biological effects of QCT on cells are dependent on the drug concentration, cell type, and exposure time.^34^, ^35^ QCT is a potent inducer of apoptosis at high concentrations. The presence of hydroxyl groups in the o‐dihydroxy substituents on the B ring of QCT makes it susceptible to autooxidation or enzymatic oxidation to form toxic o‐semiquinone and o‐quinone/quinone methylation intermediates, which can generate free radicals that lead to oxidative damage to nucleic acids, lipid peroxidation, and cell death, thereby resulting in cellular toxicity.^36^, ^37^, ^38^ In prostate cancer, 40 μM QCT affects mitochondrial integrity and promotes ROS production, leading to PCa cell death.^39^ Other studies demonstrated that high concentrations of QCT induced endoplasmic reticulum stress via the p‐STAT3/Bcl‐2 axis in ovarian cancer cells, inducing apoptosis.^40^ Therefore, QCT acts as a double‐edged sword in cells. Current knowledge of its therapeutic potential is primarily based on in vitro experiments. More research is needed through comprehensive in vivo studies to understand its pharmacokinetics, safe and effective dosages, and potential toxic side effects.

However, this study had some limitations owing to time and financial constraints. First, the mechanism by which QCT inhibits miR‐149‐3p expression was not explored. Second, the regulatory pathways downstream of AKT1 in trophoblasts have not been investigated. Then, no in vivo experiments were performed to verify our hypothesis or for safety assessment. Further research is required to translate the research findings into clinical applications.

## 5 CONCLUSION

Our study was the first to demonstrate that QCT increases HTR‐8/SVneo cell viability, promotes cell migration and invasion, and reduces apoptosis by regulating the miR‐149‐3p/AKT1 axis. Understanding the mechanism of action of QCT may facilitate the development of targeted therapies for RSA.

## CONFLICT OF INTEREST STATEMENT

The authors declare no conflicts of interest.
